# How I monitor cerebral autoregulation

**DOI:** 10.1186/s13054-019-2454-1

**Published:** 2019-05-07

**Authors:** Samuel P. Klein, Bart Depreitere, Geert Meyfroidt

**Affiliations:** 1 Department of Neurosurgery, University Hospitals Leuven, Leuven, Belgium; 2 Department of Intensive Care Medicine, University Hospitals Leuven, Leuven, Belgium

**Keywords:** Cerebrovascular autoregulation, Monitoring, Brain, Cerebral blood flow, Cerebral perfusion pressure

Cerebrovascular pressure autoregulation (CAR) protects the brain against changes in cerebral perfusion pressure (CPP) by adjusting the vascular resistance, to ensure a steady cerebral blood flow (CBF). The role of impaired CAR is well-described in the pathophysiology of traumatic brain injury (TBI), stroke, subarachnoid hemorrhage (SAH), and prematurity-related intracranial hemorrhage [1], but also in sepsis-associated brain dysfunction [2]. A clinical tool to assess CAR in real time may improve our understanding of the role of disturbed CBF in brain injury and systemic insults and open the door for personalized arterial blood pressure (ABP) manipulation to remain within the limits of active CAR [3].

To evaluate CAR, CBF changes in response to induced or spontaneous CPP fluctuations have to be analyzed. Intact CAR implies that changes in CPP will not be reflected in CBF changes. Direct assessment of global CBF is a challenge, while regional CBF can be assessed with a laser Doppler flow (LDF) probe. For the purpose of CAR monitoring, surrogate signals are often used (Fig. 1), such as intracranial pressure (ICP), or brain tissue oxygenation (PbtO_2_). Because these techniques involve inserting a probe in the brain, they cannot be applied in non-brain injury, for instance in sepsis or during anesthesia. Interestingly, and most promising, non-invasive signals that react on CBF changes can also be used, such as transcranial Doppler (TCD) or regional cerebral oxygenation as measured by near-infrared spectroscopy [2, 4].

**Fig. 1:**
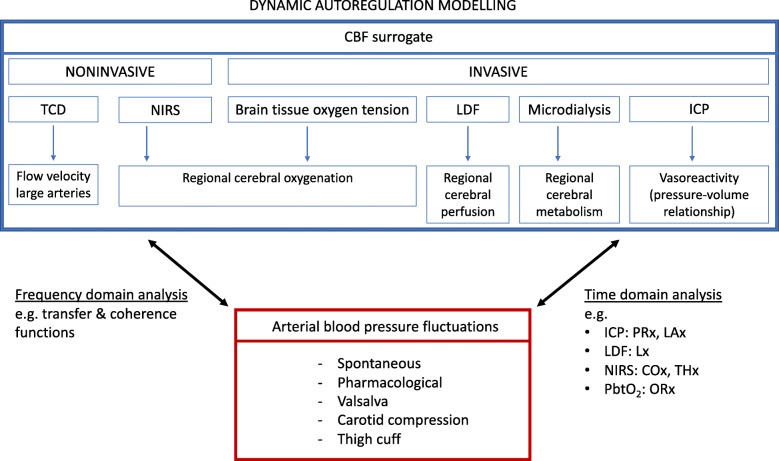
Dynamic autoregulation modeling. CBF, cerebral blood flow; TCD, transcranial Doppler; NIRS, near-infrared spectroscopy; LDF, laser Doppler flow; ICP, intracranial pressure; PRx, pressure reactivity index; LAx, low-frequency autoregulation index, Mx, TCD-based reactivity index between cerebral blood flow velocity and arterial blood pressure; Lx, autoregulation index based on LDF; COx, cerebral oxygenation index; THx, total hemoglobin index; PbtO_2_, brain tissue oxygenation; ORx, oxygen reactivity index

A distinction is made between static and dynamic CAR. Static CAR is assessed by analyzing changes in CBF in response to an ABP challenge, over a timescale of minutes (to hours), and reflects a steady-state response. Dynamic CAR does not require such formal CPP challenge, but analyzes changes in CBF in response to spontaneous CPP fluctuations over seconds or even heartbeats, by using computational techniques. Linear and nonlinear methods, using either time or frequency domain analysis, have been developed to evaluate this pressure-flow relationship [5]. The lower signal-to-noise ratio is compensated by performing multiple sequential estimates. There is a good correlation between measures of static and dynamic CAR [6]. In clinical practice, we use both.

When patients are on the steep part of the intracranial volume-pressure curve, sudden changes in cerebral blood volume induced by CBF are reflected as changes in ICP due to the reduced compliance in the rigid skull, as described by the Monro-Kellie hypothesis. This can be exploited in a dynamic as well as a static way to assess CAR. The pressure reactivity index (PRx) is based on high-frequency moving time series Pearson correlations between ICP and slow wave fluctuations of arterial blood pressure (ABP) and is available as a software package (ICM+®, Cambridge Enterprise, University of Cambridge, UK) that is frequently used for this purpose [7], including in our center. PRx thresholds for active and passive CAR have been based on retrospective studies [8] and may vary. Roughly, a more positive PRx indicates passive pressure reactivity (when a rise in ABP leads to passive vasodilation with an increased intracranial volume), while a negative or close to zero PRx indicates intact pressure reactivity. ICM+® is able to use other signals, such as PbtO_2_ for the oxygen reactivity index (ORx) [9], or a non-invasive signal such as the TCD measured CBF velocity (CBFV) for the mean velocity index (Mx) [10] or the systolic flow index (Sx) [11]. However, and importantly, the ICM+® software is currently not licensed as a medical device and should be considered research software. When high-frequency waveform data are not available, which is typically the case in most patient data management systems, the low-frequency autoregulation index (LAx) can be used. LAx uses minute-by-minute ABP-ICP correlations to calculate CAR [12].

A very elegant technique to assess static CAR at the bedside that does not require additional software and can be used in all patients where a surrogate CBF signal is being monitored, in a single or multimodal way, as described above, is the application of an ABP challenge. In this procedure, the mean ABP is raised 10 mmHg by increasing the vasopressor dose, while assessing ICP, PbtO_2_, and/or CBFV, after 10–20 min. A decrease, or no change, in ICP, indicates intact CAR, while an increase in ICP demonstrates the absence of CAR. Inherently, a successful ABP challenge indicating intact CAR will increase CPP. Typically, in response to this higher CPP, PbtO_2_ will improve. CBFV changes can be interpreted in the same way (should remain constant or increase when CAR is intact, should decrease because of vasodilation when CAR is impaired). When brain hyperperfusion is suspected, a reverse ABP challenge, where ABP is decreased by 10 mmHg, can also be considered. In our ABP challenge protocol, we are cautious when going outside CPP ranges of 60–80 mmHg, and will never recommend CPP outside 50–90 mmHg.

In summary, it is important to realize that no ‘gold-standard’ clinical CAR monitor exists, and basic physiological research to support the theory behind different monitoring techniques and improve our understanding is highly needed [13]. Our current CAR monitoring protocol is based on the multimodal combination of different static and dynamic measures. Severe TBI patients, for instance, are routinely monitored with an intraparenchymal ICP probe, and in selected patients, a PbtO_2_ probe is added. Dynamic CAR is monitored using ICM+® software, recognizing the limitations described above [14]. We consider an ABP challenge as second-tier therapy for isolated elevated ICP, or for intracranial hypertension combined with low PbtO_2_, only after correction of other first-tier level factors [15]. In these cases, when ICM+® indicates that the zone of optimal CPP might lie outside the 60–70 range, this can be interpreted as a clinical predictor of a potentially successful ABP challenge. This relatively simple test can be performed by an experienced intensivist, at the bedside, provided caution is applied when leaving the CPP range of 60–80 mmHg, and provided the situation is re-evaluated frequently. Currently, there is insufficient evidence to advice the use of one single bedside monitoring technique.

## Abbreviations

ABP: Arterial blood pressure
CAR: Cerebrovascular pressure autoregulation
CBF: Cerebral blood flow
CBFV: Cerebral blood flow velocity
COx: Cerebral oxygenation index
CPP: Cerebral perfusion pressure
ICM: Intensive Care Monitor plus software
ICP: Intracranial pressure
LAx: Low-frequency autoregulation index
LDF: Laser Doppler flow
Lx: Autoregulation index based on LDF
Mx: Mean arterial blood flow velocity index
ORx: Oxygen reactivity index
PbtO_2_: Brain tissue oxygenation
PRx: Pressure reactivity index
SAH: Subarachnoid hemorrhage
Sx: Systolic blood flow velocity index
TBI: Traumatic brain injury
TCD: Transcranial Doppler
THx: Total hemoglobin index
